# ﻿New species and records of the genus *Antocha* Osten Sacken (Diptera, Limoniidae) from Tibet, China with a key to species in Qinghai-Tibet region

**DOI:** 10.3897/zookeys.1156.86786

**Published:** 2023-03-24

**Authors:** Hanhuiying Lv, Juan Sun, Ning Wang, Ding Yang, Xiao Zhang

**Affiliations:** 1 Shandong Engineering Research Center for Environment-Friendly Agricultural Pest Management, College of Plant Health and Medicine/College of Grassland Science, Qingdao Agricultural University, Qingdao 266109, China Key Laboratory of Biohazard Monitoring and Green Prevention and Control in Artificial Grassland, Ministry of Agriculture and Rural Affairs, Institute of Grassland Research, Chinese Academy of Agricultural Sciences Hohhot China; 2 Key Laboratory of Biohazard Monitoring and Green Prevention and Control in Artificial Grassland, Ministry of Agriculture and Rural Affairs, Institute of Grassland Research, Chinese Academy of Agricultural Sciences, Hohhot 010010, China Qingdao Agricultural University Qingdao China; 3 College of Plant Protection, China Agricultural University, Beijing 100193, China China Agricultural University Beijing China

**Keywords:** Chinese fauna, crane flies, Limoniinae, Qinghai-Tibet Plateau, taxonomy

## Abstract

Thirty-four known species and subspecies of the genus *Antocha* Osten Sacken, 1860 have been recorded from China, of which four occur in Tibet. Herein, two new *Antocha* species, A. (Antocha) curvativa**sp. nov.** and A. (A.) tibetana**sp. nov.**, are described and illustrated from Tibet. The new species are distinguished from congeners mainly by their male genitalia. Antocha (A.) spiralis Alexander, 1932 and A. (A.) setigera Alexander, 1933, which are newly recorded in Tibet, are redescribed and illustrated. A key to *Antocha* species in the Qinghai-Tibet region of China is also presented.

## ﻿Introduction

The genus *Antocha* Osten Sacken, 1860 is a medium-sized genus of 161 described species and subspecies in the family Limoniidae (Oosterbroek 2023). It is known from the Oriental (83 species and subspecies), Palaearctic (56 species and subspecies), Afrotropic (21 species), Nearctic (seven species), Australasian (three species), and Neotropic (one species) regions (Oosterbroek 2023). A conspicuous feature of the genus is that the anal angle of the wing is nearly right-angled, and detailed features for the recognition of the genus were given by Osten Sacken (1860), Alexander (1968), and Markevičiūtė et al. (2019, 2021). In the past three decades, many taxonomic studies have been carried out on Asian *Antocha*, mainly focusing on the species in Japan (Torii 1992a, 1992b, 1992c, 1996), China (Podenas and Young 2015; Markevičiūtė et al. 2019, 2021), South Korea (Podenas and Byun 2014), North Korea (Podenas 2015), and Indonesia (Young 1994).

Tibet is located in the Qinghai-Tibet region of China, which also includes all of Qinghai, western Sichuan, and small parts of Gansu, Xinjiang, and Yunnan. The main body of the region is the Qinghai-Tibet Plateau, which is known as the “roof of the world” because of its high terrain and extensive grasslands. The Qinghai-Tibet region is also the source of many rivers in China.

**Figure 1.**
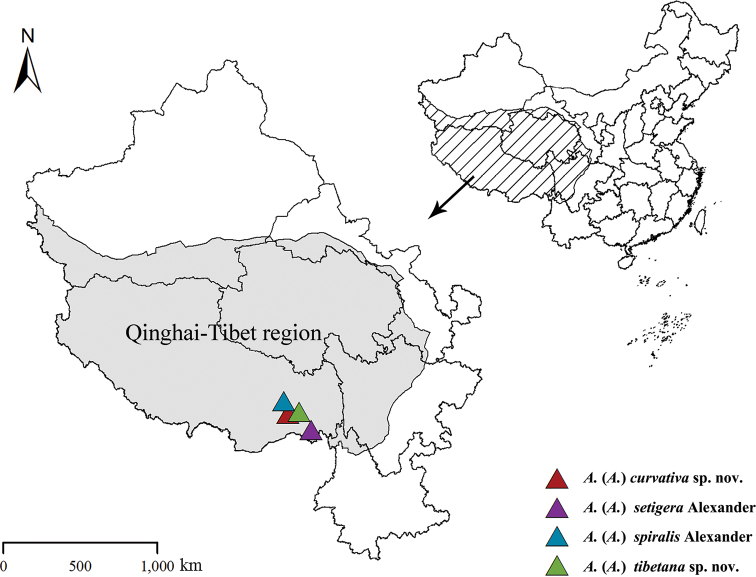
Collecting sites of *Antocha* species in China.

At present, 34 *Antocha* species and subspecies are recorded from China, of which 22 are known in the Qinghai-Tibet region, while only four are distributed in Tibet (Oosterbroek 2023). In this study, specimens of *Antocha* from Tibet have been examined, and four species are added to the fauna of Tibet (Fig. 1), of which A. (A.) curvativa sp. nov. and A. (A.) tibetana sp. nov. are described and illustrated as new to science, and A. (A.) spiralis Alexander, 1932 and A. (A.) setigera Alexander, 1933 are newly recorded from Tibet. More comprehensive redescriptions and illustrations for the two known species, as well as a key to the *Antocha* crane flies in Qinghai-Tibet region, are also presented.

## ﻿Materials and methods

All specimens for this study were collected from Tibet, China by various entomologists in 2014–2018. Type specimens are deposited in Entomological Museum of China Agricultural University, Beijing, China (**CAU**). The holotype of A. (A.) setigera, deposited in the National Museum of Natural History, Smithsonian Institution, Washington, DC, USA (**USNM**), was also examined. Genitalic preparations of males were made by macerating the apical portion of the abdomen in cold 10% hydroxide (NaOH) for 12–15 hours. Observations and illustrations were made using a ZEISS Stemi 2000-C stereomicroscope. Photographs were taken with a Canon EOS 90D digital camera through a macro lens. Details of coloration were examined in specimens immersed in 75% ethanol (C_2_H_5_OH).

The morphological terminology mainly follows Cumming and Wood (2017) and de Jong for wing venation (2017). The term “inner branch of paramere” is adopted from Kato and Tachi (2019). The general distribution of species is given according to Oosterbroek (2023).

The following abbreviations in figures are used: aed = aedeagus, app = apical part of paramere, bp = base of paramere, cerc = cercus, goncx = gonocoxite, hyp vlv = hypogynial valve, i gonst = inner gonostylus, ib = interbase, ibp = inner branch of paramere, o gonst = outer gonostylus, pm = paramere, tg 9 = tergite 9,tg 10 = tergite 10.

## ﻿Checklist of *Antocha* crane flies in Qinghai-Tibet region of China

New province records in bold

Antocha (Antocha) bella Markevičiūtė & Podenas, 2019 (Sichuan)

Antocha (Antocha) bidens Alexander, 1932 (Sichuan)

Antocha (Antocha) bifida Alexander, 1924 (Sichuan, Guangdong, Taiwan; Russia; Kazakhstan; Mongolia; North Korea; South Korea; Japan; Philippines)

Antocha (Antocha) constricta Alexander, 1932 (Sichuan)

Antocha (Antocha) curvativa Lv & Zhang sp. nov. (Tibet)

Antocha (Antocha) emarginata Alexander, 1938 (Sichuan)

Antocha (Antocha) flavidibasis Alexander, 1938(Sichuan)

Antocha (Antocha) fortidens Alexander, 1933 (Sichuan, Tibet)

Antocha (Antocha) indica Brunetti, 1912 (Sichuan, Zhejiang; India; Malaysia)

Antocha (Antocha) lacteibasis Alexander, 1935 (Sichuan)

Antocha (Antocha) minuticornis Alexander, 1931 (Sichuan)

Antocha (Antocha) multidentata Alexander, 1932 (Sichuan)

Antocha (Antocha) nebulipennis immaculata Alexander, 1938 (Sichuan; Myanmar)

Antocha (Antocha) nebulipennis nebulipennis Alexander, 1931 (Gansu, Sichuan, Tibet; India; Nepal; Tajikistan; Afghanistan)

Antocha (Antocha) nigribasis Alexander, 1932 (Sichuan)

Antocha (Antocha) pallidella Alexander, 1933 (Sichuan)

Antocha (Antocha) picturata Alexander, 1936 (Sichuan)

Antocha (Antocha) pterographa Alexander, 1953 (Tibet)

Antocha (Antocha) pulchra Markevičiūtė & Podenas, 2021 (Sichuan)

Antocha (Antocha) quadrifurca Alexander, 1971 (Sichuan; India)

Antocha (Antocha) setigera Alexander, 1933 (Sichuan, **Tibet**)

Antocha (Antocha) spiralis Alexander, 1932 (Sichuan, **Tibet**; India)

Antocha (Antocha) tibetana Lv & Zhang sp. nov. (Tibet)

Antocha (Antocha) yatungensis Alexander, 1963 (Tibet)

## ﻿Taxonomy

### ﻿Key to *Antocha* species from Qinghai-Tibet region of China

**Table:** 

1	Wing with distinct brown or dark gray stigma	**2**
–	Wing without stigma or with indistinct stigma (Figs 2d, 4d, 6d, 8d)	**9**
2	Crossvein m-cu long before fork of M, distance about its own length	**3**
–	Crossvein m-cu at or short before fork of M, distance less than half of own length (Figs 2d, 4d, 6d, 8d)	**4**
3	Prescutum and presutural scutum uniformly light yellow. Crossvein m-cu more than one and half times its own length, before fork of M. Posterior margin of tergite 9 with median emarginate (Alexander 1932; Markevičiūtė et al. 2019, 2021)	**A. (A.) nigribasis**
–	Prescutum and presutural scutum grey with three brown stripes. Crossvein m-cu a little less than its own length, before fork of M. Posterior margin of tergite 9 without median emarginate (Alexander 1953)	**A. (A.) pterographa**
4	Cell m_1_ longer than cell dm (Figs 4d, 6d, 8d)	**5**
–	Cell m_1_ almost as long as or shorter than cell dm (Fig. 2d)	**6**
5	Prescutum and presutural scutum grey, with a brown stripe. Vein Sc ending nearly fork of Rs (Alexander 1936)	**A. (A.) picturata**
–	Prescutum and presutural scutum uniformly yellowish gray, without stripes. Vein Sc ending a greater distance before fork of Rs (Alexander 1931; Markevičiūtė et al. 2019, 2021)	**A. (A.) nebulipennis nebulipennis**
6	Apex of outer gonostylus not bifid	**7**
–	Apex of outer gonostylus bifid	**8**
7	Inner branch of paramere with outer tooth shorter than inner tooth (Alexander 1932; Markevičiūtė et al. 2019)	**A. (A.) multidentata**
–	Inner branch of paramere with outer tooth longer than inner tooth (Alexander 1971; Markevičiūtė et al. 2021)	**A. (A.) quadrifurca**
8	Posterior margin of tergite 9 with two small lobes far away from each other, middle flat (Alexander 1932; Markevičiūtė et al. 2019, 2021)	**A. (A.) bidens**
–	Posterior margin of tergite 9 with two close lobes, middle concave (Alexander 1933a; Markevičiūtė et al. 2019, 2021)	**A. (A.) fortidens**
9	Crossvein m-cu long before fork of M, distance about its own length	**10**
–	Crossvein m-cu at or short before fork of M, distance less than half of its own length (Figs 2d, 4d, 6d, 8d)	**11**
10	Basal section of R_5_ slightly longer than r-m. Posterior margin of tergite 9 with two small lobes, middle flat (Alexander 1933b; Markevičiūtė et al. 2021)	**A. (A.) pallidella**
–	Basal section of R_5_ nearly twice as long as r-m. Posterior margin of tergite 9 with two big lobes, middle concave (Alexander 1963)	**A. (A.) yatungensis**
11	Vein Sc ending before fork of Rs (Figs 2d, 6d, 8d)	**12**
–	Vein Sc ending at or beyond fork of Rs (Fig. 4d)	**19**
12	Prescutum and presutural scutum without stripe (Fig. 6c)	**13**
–	Prescutum and presutural scutum with stripe(s) (Figs 2c, 4c, 8c)	**14**
13	Apical part of paramere with three small branches (Alexander 1932; Markevičiūtė et al. 2019, 2021)	**A. (A.) constricta**
–	Apical part of paramere slender and twisted into spiral, without branches (Figs 6e, 7)	**A. (A.) spiralis**
14	Antennae with scape yellow, remaining segments black	**15**
–	Antennae black, dark brown, or brown throughout (Figs 2b, 8b)	**16**
15	Basal section of M_3_ as long as m-m. Posterior margin of tergite 9 flat. Inner gonostylus narrowed to obtuse tip (Brunetti 1912; Markevičiūtė et al. 2019, 2021)	**A. (A.) indica**
–	Basal section of M_3_ twice as long as m-m. Posterior margin of tergite 9 with two rounded lobes. Inner gonostylus with tip dilated (Alexander 1938a; Markevičiūtė et al. 2019, 2021)	**A. (A.) nebulipennis immaculata**
16	Basal section of M_3_ shorter than one and half times length of m-m (Alexander 1938a)	**A. (A.) flavidibasis**
–	Basal section of M_3_ as long as or longer than twice length of m-m (Figs 2d, 8d)	**17**
17	Tip of inner branch of paramere bifid (Figs 8e, 9)	**A. (A.) tibetana sp. nov.**
–	Tip of inner branch of paramere not bifid (Figs 2e, 3, 4e, 5)	**18**
18	Basal section of M_3_ four times as long as m-m (Fig. 2d). Outer gonostylus with tip inflated and blunt (Figs 2e, 3)	**A. (A.) curvativa sp. nov.**
–	Basal section of M_3_ twice as long as m-m. Outer gonostylus narrowed to acute tip (Alexander 1935; Markevičiūtė et al. 2019, 2021)	**A. (A.) lacteibasis**
19	Tip of outer gonostylus bifid (Markevičiūtė et al. 2021: Figs 10, 15)	**20**
–	Tip of outer gonostylus not bifid	**21**
20	Posterior margin of tergite 9 with two teeth (Alexander 1924; Markevičiūtė et al. 2019, 2021)	**A. (A.) bifida**
–	Posterior margin of tergite 9 without teeth (Markevičiūtė et al. 2021)	**A. (A.) pulchra**
21	Inner branch of paramere spiral (Markevičiūtė et al. 2019, 2021)	**A. (A.) bella**
–	Inner branch of paramere straight (Figs 4e, 5)	**22**
22	Posterior margin of tergite 9 with a deep, U-shaped, median concavity (Alexander 1938b; Markevičiūtė et al 2019, 2021)	**A. (A.) emarginata**
–	Posterior margin of tergite 9 with a gentle median concavity (Fig. 5)	**23**
23	Tip of aedeagus not bifid (Alexander 1931; Markevičiūtė et al. 2019, 2021)	**A. (A.) minuticornis**
–	Tip of aedeagus bifid (Figs 4e, 5)	**A. (A.) setigera**

#### ﻿ Antocha (Antocha) curvativa

Taxon classificationAnimalia

Lv & Zhang sp. nov.

61E00583-E9D7-5C20-A801-081D27980182

https://zoobank.org/CE9CC4CC-B440-40E5-979D-35F2A4843FEB

Figs 2 , 3

##### Type material.

***Holotype***: China • ♂; Tibet Autonomous Region, Medog County, Bari village; 29°20'13"N, 95°21'54"E; 1680 m a.s.l.; 29 July 2014; Yan Li leg; CAU. ***Paratypes***: China • 2 ♂♂ 2 ♀♀; same data as holotype; CAU.

##### Diagnosis.

Antocha (A.) curvativa sp. nov. can be recognized by thorax with four more or less confluent stripes, wing having no stigma, basal section of M_3_ which is about four times as long as m-m, posterior margin of tergite 9 having shallow median emargination and specific, stout outer gonostylus with tip distinctly flattened and nearly funnel-shaped. Aedeagal complex with interbase elongated, distally oval; paramere apically slender and curved ventrally; inner branch of paramere elongated, tip rounded.

##### Description.

**Male.** Body length 4.8–5.5 mm, wing length 4.3–4.8 mm, antenna length 0.9–1.1 mm.

**Figure 2.**
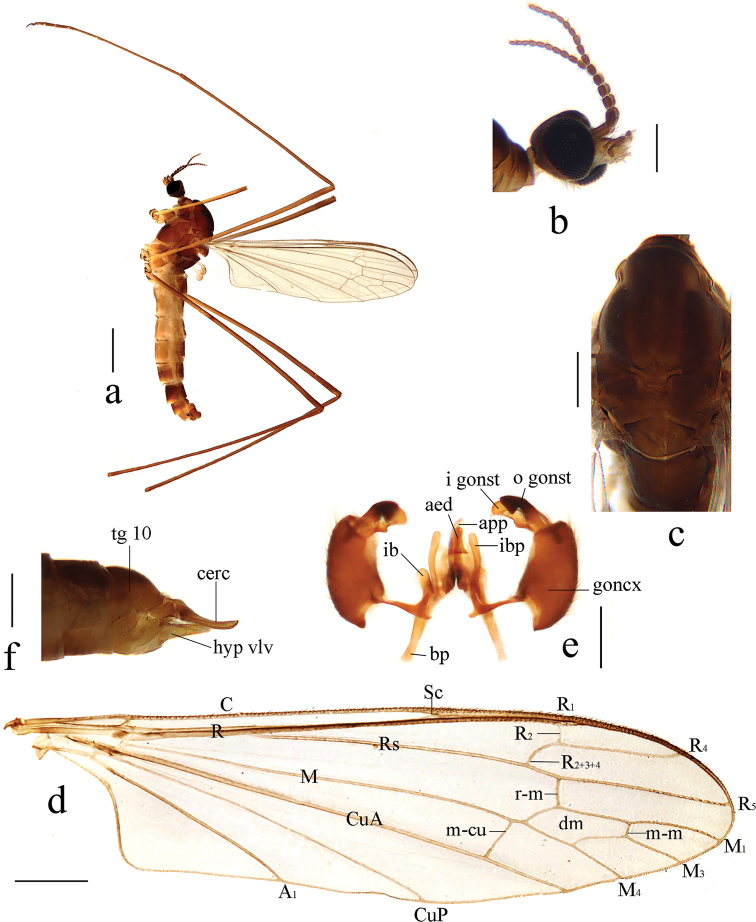
Antocha (Antocha) curvativa sp. nov. **a** habitus of male, lateral view **b** male head, lateral view **c** male thorax, dorsal view **d** male wing **e** aedeagal complex with gonocoxite and gonostyli, dorsal view **f** female ovipositor, lateral view. Scale bars: 1.0 mm (**a**); 0.3 mm (**b, c**); 0.5 mm (**d**); 0.2 mm (**e, f**).

***Head*** (Fig. 2b). Dark brown, with brown setae. Antenna dark brown. Scape cylindrical; pedicel oval; flagellomeres oval, apically tapering and shortened. Setae on antenna brown. Rostrum light brown; palpus brown to dark brown; setae on rostrum and palpus brown.

***Thorax*** (Fig. 2c). Pronotum dark brown. Prescutum and presutural scutum brown, with four more or less confluent dark brown stripes. Postsutural scutum brown; scutal lobes each with a darker brown spot. Scutellum brown, with side edges dark brown. Mediotergite dark brown. Pleuron brown (Fig. 2a). Legs with light brown coxae; trochanters light yellow with side edges brown; femora yellowish, becoming brown towards apex; tibiae and tarsal segments brown. Setae on legs brown. Wing light brown, without stigma; anal angle nearly right-angled (Fig. 2d). Veins light brown. Venation: Sc ending before fork of Rs, at about 2/3 of Rs; basal section of R_5_ about as long as r-m; m-cu shortly before fork of M, distance approximately 1/4 its own length; basal section of M_3_ about four times as long as m-m; cell m_1_ about as long as cell dm. Halter with stem pale.

***Abdomen*.** Tergites 1–6 brown, tergites 7 and 8 dark brown. Sternites 1–6 light brown, sternites 7 and 8 dark brown.

**Figure 3.**
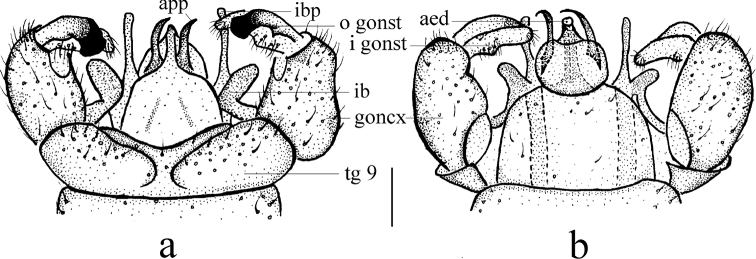
Antocha (Antocha) curvativa sp. nov. **a** male hypopygium, dorsal view **b** male hypopygium, ventral view. Scale bar: 0.1 mm.

***Hypopygium*** (Figs 2e, 3). Brown. Posterior margin of tergite 9 with broad and shallow emargination (Fig. 3a). Gonocoxite nearly cylindrical, with long yellow setae (Figs 2e, 3). Outer gonostylus stout, apical half sclerotized, tip distinctly curved, flattened, nearly funnel-shaped. Inner gonostylus thick and fleshy. Interbase nearly V-shaped, distal part elongate and oval (Figs 2e, 3). Paramere with base rod-shaped, apical part slender, curved ventrally, and with tip sharp. Inner branch of paramere in the shape of elongated lobe with tip rounded (Figs 2e, 3). Aedeagus rod-shaped, curved ventrally (Figs 2e, 3).

**Female.** Body length 5.0–5.5 mm, wing length 4.5–5.0 mm. Generally similar to male by body coloration.

***Ovipositor*** (Fig. 2f). Tergite 10 dark brown, caudal part paler. Cercus brown with base darker, tip raised and tapering. Hypogynial valve light brown, reaching approximately 3/5 of cercus.

##### Etymology.

The specific name refers to the curved apical part of paramere.

##### Distribution.

China (Tibet).

##### Remarks.

The new species is similar to A. (A.) lacteibasis from China in having similar apical part of paramere and tip of inner branch of paramere being not bifid, but it can be easily distinguished by the basal section of vein M_3_ being about four times as long as m-m (Fig. 2d) and the stout outer gonostylus with the tip sclerotized, funnel-shaped, inflated, and blunt (Figs 2e, 3). In A. (A.) lacteibasis, the basal section of vein M_3_ is about twice as long as m-m, and the outer gonostylus narrow with acute tip (Alexander 1935; Markevičiūtė et al. 2019, 2021).

#### ﻿ Antocha (Antocha) setigera

Taxon classificationAnimalia

Alexander

1F09FB63-1937-5676-B812-11C5E315BEAD

Figs 4 , 5

Antocha (Antocha) setigera Alexander 1933b: 369 (original description).

##### Type material examined.

***Holotype***: China • ♂; Sichuan province, Mount Omei; 2134 m a.s.l.; 17 July 1931; Franck leg; USNM. **Other material examined**: China • 8 ♂♂ 6 ♀♀; Tibet Autonomous Region, Chayu County, Xiachayu Farm Hydropower Station; 28°30'19"N, 97°01'25"E; 1520 m a.s.l.; 8 July 2016; Shaolin Han leg; CAU.

##### Diagnosis.

Antocha (A.) setigera can be recognized by thorax with four brown stripes, wing lacking a stigma, basal section of M_3_ as long as m-m, and slightly curved outer gonostylus with blackened, blunt tip. Aedeagal complex with interbase distally small and parameres apically fused, arch-shaped.

##### Description.

**Male.** Body length 4.5–5.0 mm, wing length 5.0–6.0 mm, antenna length 1.5–1.8 mm.

**Figure 4.**
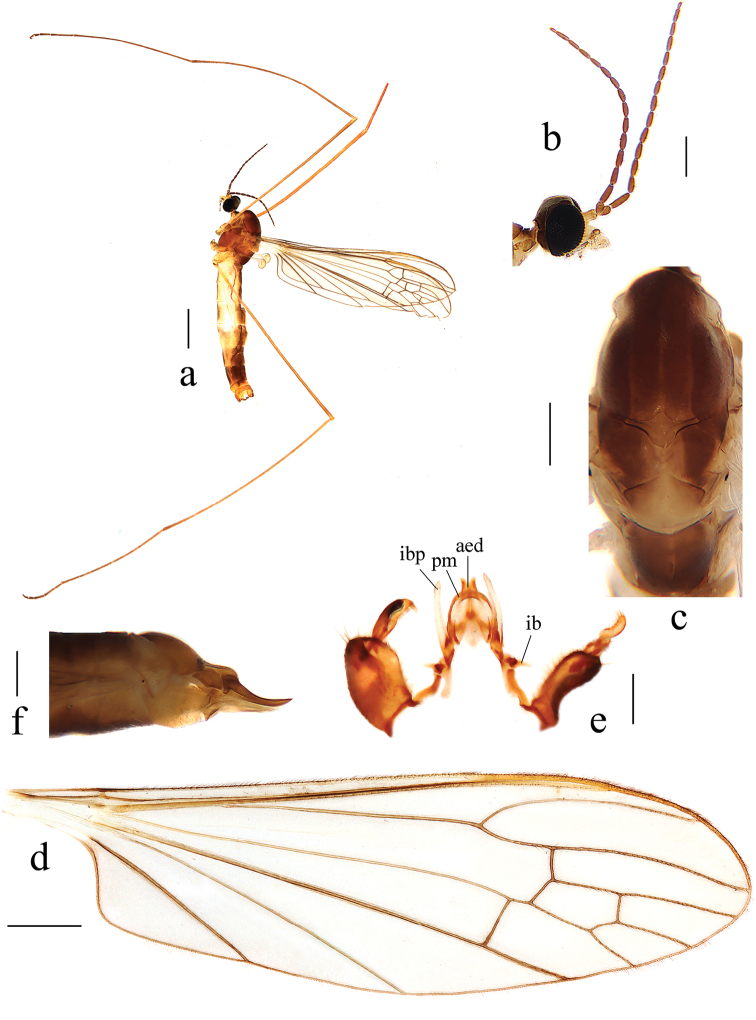
Antocha (Antocha) setigera**a** habitus of male, lateral view **b** male head, lateral view **c** male thorax, dorsal view **d** male wing **e** aedeagus complex with gonocoxite and gonostyli, dorsal view **f** female ovipositor, lateral view. Scale bars: 1.0 mm (**a**); 0.2 mm (**b, c**); 0.5 mm (**d**); 0.2 mm (**e, f**).

***Head*** (Fig. 4b). Dark brown with brown setae. Antenna brown with light brown scape. Scape cylindrical; pedicel and flagellomeres elongate oval, apically tapering; terminal segment short, about half as short as other segments. Rostrum yellow; palpus light brown; setae on rostrum and palpus brown.

***Thorax*** (Fig. 4c). Pronotum brown. Prescutum and presutural scutum brown four brown stripes. The central stripes fused in the anterior third, the rest is separated by pale narrow vitta. Postsutural scutum dark brown, middle area yellow, scutal lobes each with brown spot. Scutellum pale yellow, with side edges dark brown. Mediotergite brown to dark brown. Pleuron brown (Fig. 4a). Legs with fore coxa brown; mid coxa brownish yellow; hind coxa yellow; trochanters yellow; femora and tibiae brownish yellow; tarsi brown with terminal segments darker brown. Wing light brown, without stigma; anal angle nearly right-angled (Fig. 4d). Veins brown. Venation: Sc ending nearly at fork of Rs; basal section of R_5_ about twice as long as r-m; m-cu shortly before fork of M, distance approximately 1/3 its own length; basal section of M_3_ as long as m-m; cell m_1_ longer than cell dm. Halter pale with stem light yellow.

***Abdomen*.** Tergites dark brown. Sternites 1–6 brown with side edges yellow, sternites 7 and 8 dark brown.

**Figure 5.**
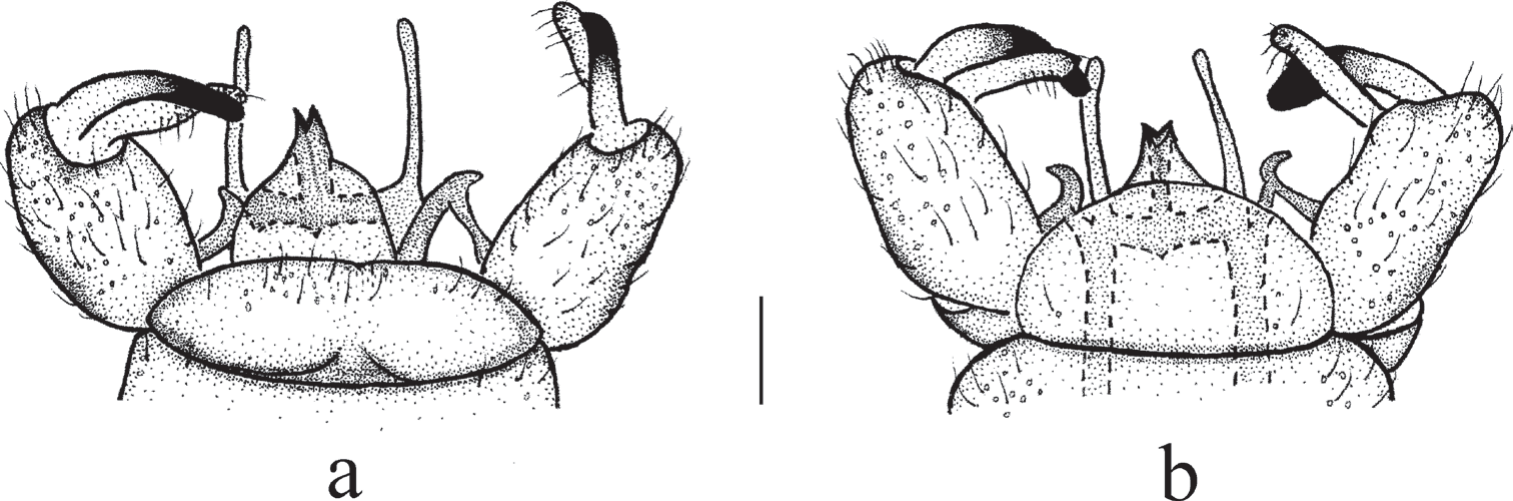
Antocha (Antocha) setigera**a** male hypopygium, dorsal view **b** male hypopygium, ventral view. Scale bar: 0.1 mm.

***Hypopygium*** (Figs 4e, 5). Yellow. Posterior margin of tergite 9 convex with middle slightly emarginate (Fig. 5a). Gonocoxite nearly cylindrical with long brown setae (Figs 4e, 5). Outer gonostylus slightly curved, with blackened distal half and tip sclerotized and blunt. Inner gonostylus slightly curved. Interbase distally flattened, small and horn-like with tip sharp (Figs 4e, 5). Parameres apically fused, arch-shaped. Inner branch of paramere elongated, with tip narrowly obtuse (Figs 4e, 5). Aedeagus curved ventrally, tip bifid (Figs 4e, 5).

**Female.** Body length 4.5–5.5 mm, wing length 5.0–6.0 mm. Generally similar to male by body coloration.

***Ovipositor*** (Fig. 4f). Tergite 10 yellowish brown with base darker. Cercus brown, tip raised and tapering, apex acute. Hypogynial valve yellowish, reaching sub-tip of cercus.

##### Distribution.

China (Sichuan, Tibet).

##### Remarks.

In China, this species was previously only known in Sichuan province and is now recorded in Tibet for the first time. For descriptions and illustrations of this species, also see Alexander (1933b) and Markevičiūtė et al. (2019, 2021).

#### ﻿ Antocha (Antocha) spiralis

Taxon classificationAnimalia

Alexander

B06A36D1-F7C3-5E45-8623-85090E10E985

Figs 6 , 7

Antocha (Antocha) spiralis Alexander 1932: 389 (original description).

##### Material examined.

China • 9 ♂♂ 1 ♀; Tibet Autonomous Region, Bayi District, Pailong; 30°01'25"N, 95°00'32"E; 2003 m a.s.l.; 20 June 2018; Liang Wang leg.; CAU.

##### Diagnosis.

Antocha (A.) spiralis can be recognized by thorax having no stripes, wing without stigma, basal section of M_3_ about one and half times as long as m-m, posterior margin of tergite 9 having shallow, median emargination and slightly curved, blackened in distal 2/3 of outer gonostylus. Aedeagal complex with interbase nearly U-shaped; paramere apically slender and twisted into a spiral; inner branch of paramere with bifid tip.

##### Description.

**Male.** Body length 4.5–5.5 mm, wing length 5.0–6.0 mm, antenna length 1.0–1.2 mm.

**Figure 6.**
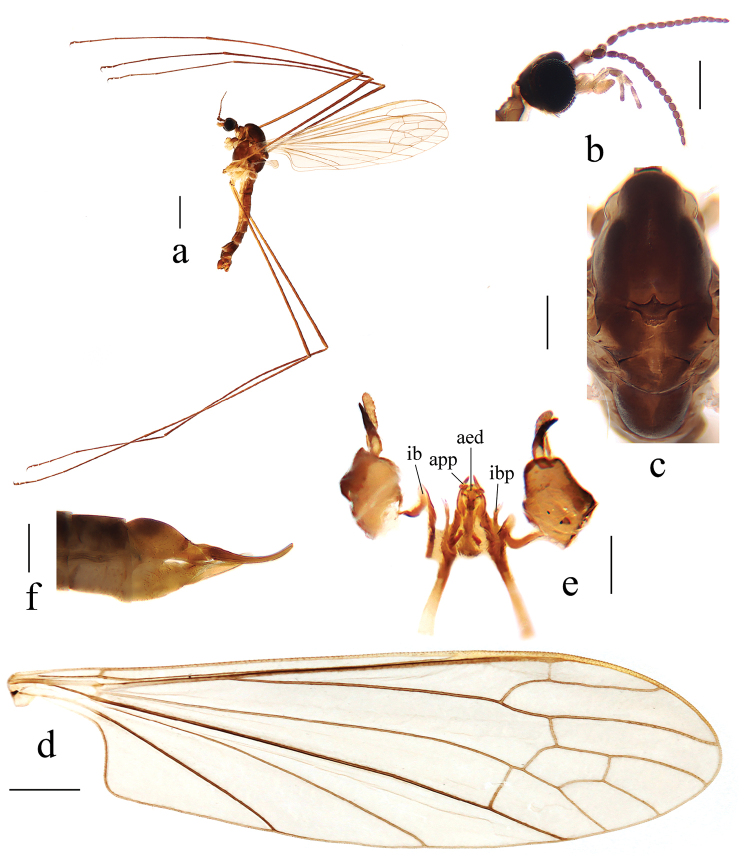
Antocha (Antocha) spiralis**a** habitus of male, lateral view **b** male head, lateral view **c** male thorax, dorsal view **d** male wing **e** aedeagus complex with gonocoxite and gonostyli, dorsal view **f** female ovipositor, lateral view. Scale bars: 1.0 mm (**a**); 0.3 mm (**b**); 0.4 mm (**c**); 0.6 mm (**d**); 0.2 mm (**e, f**).

***Head*** (Fig. 6b). Black with brown setae. Antenna brown, with dark brown pedicel. Scape cylindrical; pedicel and flagellomeres oval; terminal two segments slender. Rostrum yellow; palpus light brown; setae on rostrum and palpus brown.

***Thorax*** (Fig. 6c). Pronotum brown. Prescutum and presutural scutum dark brown, without stripe. Postsutural scutum brownish yellow in middle; scutal lobes each with brown spot. Scutellum brown with middle of base brownish yellow. Mediotergite dark brown, with middle brownish yellow. Pleuron brown (Fig. 6a). Legs with fore and mid coxae brown; hind coxa yellow; trochanters yellow with side edges brown; femora brownish yellow to brown; remaining segments dark brown. Wing light brown, without stigma; anal angle nearly right-angled (Fig. 6d). Veins brown. Venation: Sc ending before fork of Rs, at about 5/6 of Rs; basal section of R_5_ about 1½ times as long as r-m; m-cu shortly before fork of M, distance approximately 1/4 its own length; basal section of M_3_ about 1½ as long as m-m; cell m_1_ longer than cell dm. Halter pale.

***Abdomen*.** Tergites 1–6 brown, tergites 7 and 8 dark brown. Sternites 1–6 brownish yellow to light brown; sternites 7 and 8 dark brown.

**Figure 7.**
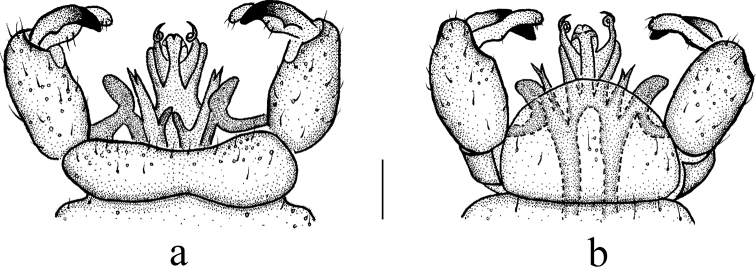
Antocha (Antocha) spiralis**a** male hypopygium, dorsal view **b** male hypopygium, ventral view. Scale bar: 0.1 mm.

***Hypopygium*** (Figs 6e, 7). Posterior margin of tergite 9 with broad and shallow emargination (Fig. 7a). Gonocoxite nearly cylindrical with brown setae (Figs 6e, 7). Outer gonostylus slightly curved; base yellowish brown; distal 2/3 blackened, narrowing towards obtuse apex. Interbase nearly U-shaped, distal part flattened and oval (Figs 6e, 7). Paramere with rod-shaped base; apical part slender and twisted into spiral; tip sharp. Inner branch of paramere with tip bifid; outer tooth longer than inner one (Figs 6e, 7). Aedeagus with two projections near tip (Figs 6e, 7).

**Female.** Body length 4.5–5.5 mm, wing length 5.0–6.0 mm. Generally similar to male by body coloration.

***Ovipositor*** (Fig. 6f). Tergite 10 brownish yellow, with brown base. Cercus yellowish brown, with base darker, slender, and curved; tip raised and tapering. Hypogynial valve yellow, reaching approximately middle of cercus.

##### Distribution.

China (Sichuan, Tibet), India.

##### Remarks.

In China, this species was previously only known in Sichuan and is now recorded in Tibet for the first time. For descriptions and illustrations of this species, also see Alexander (1932) and Markevičiūtė et al. (2019, 2021). Both A. (A.) spiralis and A. (A.) bella from China have the twisted structure of the hypopygium. In A. (A.) spiralis, the apical part of the paramere is slender and twisted into a spiral, and the tip of the inner branch of the paramere is bifid (Figs 6e, 7), while in A. (A.) bella, the tip of the inner branch of the paramere is twisted (Markevičiūtė et al. 2019).

#### ﻿ Antocha (Antocha) tibetana

Taxon classificationAnimalia

Lv & Zhang sp. nov.

0B044188-DBD1-543B-A357-3A9CB01CD496

https://zoobank.org/81CC7F50-E60F-47BD-A80C-A206C006D2FA

Figs 8 , 9

##### Type material.

***Holotype***: China • ♂; Tibet Autonomous Region, Medog County, 80k; 29°28'47"N, 96°05'19"E; 2104 m a.s.l.; 30 July 2014; Tingting Zhang leg; CAU. ***Paratypes***: China • 1 ♂ 2 ♀♀; Tibet Autonomous Region, Medog County, 80k; 29°28'47"N, 96°05'19"E; 2104 m a.s.l.; 1 Aug. 2014; Tingting Zhang leg; CAU.

##### Diagnosis.

Antocha (A.) tibetana sp. nov. can be recognized by thorax with three dark brown stripes, wing having indistinct stigma, basal section of M_3_ about twice as long as m-m, posterior margin of tergite with shallow emargination and outer gonostylus apically claw-shaped. Aedeagal complex with interbase distally horn-like; paramere apically flattened and triangular; inner branch of paramere with tip bifid into two teeth.

##### Description.

**Male.** Body length 4.5–5.0 mm, wing length 5.1–5.5 mm, antenna length 1.0–1.2 mm.

**Figure 8.**
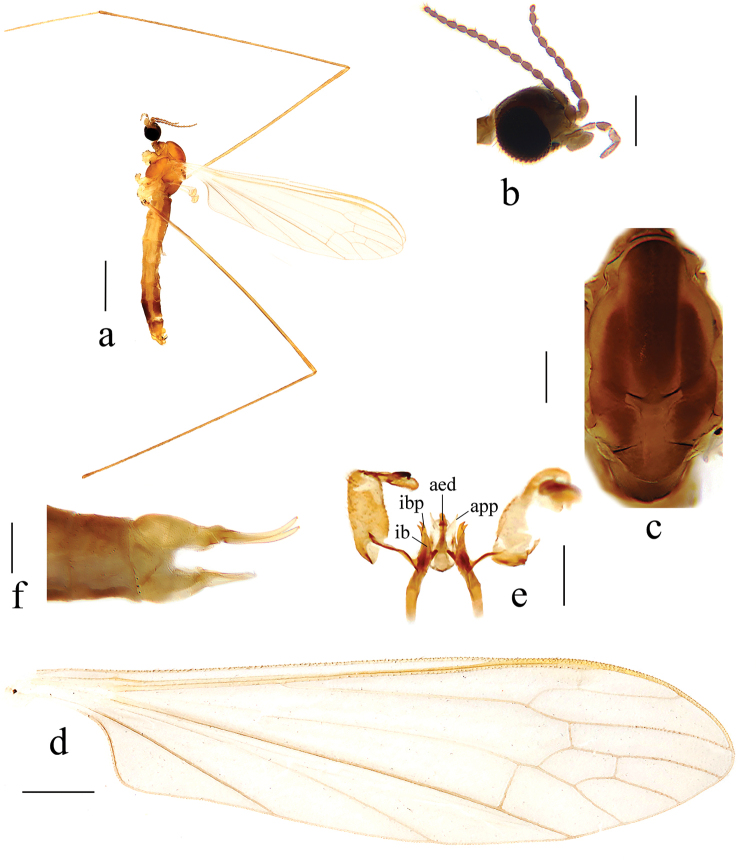
Antocha (Antocha) tibetana sp. nov. **a** habitus of male, lateral view **b** male head, lateral view **c** male thorax, dorsal view **d** male wing **e** aedeagus complex with gonocoxite and gonostyli, dorsal view **f** female ovipositor, lateral view. Scale bars: 1.0 mm (**a**); 0.3 mm (**b, c**); 0.5 mm (**d**); 0.2 mm (**e, f**).

***Head*** (Fig. 8b). Dark brown, with brown setae. Antenna brown, with brown setae. Scape nearly cylindrical; pedicel oval; flagellomeres oval, apically shortened. Rostrum and palpus brown, with brown setae.

***Thorax*** (Fig. 8c). Pronotum brown. Prescutum and presutural scutum brownish yellow, with three dark brown stripes. Postsutural scutum brownish yellow; scutal lobes each with a brown spot. Scutellum brown, with middle brownish yellow. Mediotergite brown with side edges light brown. Pleuron brownish yellow (Fig. 8a). Legs with coxae and trochanters yellow; rest of segments brownish yellow. Wing light brownish yellow, with very indistinct stigma; anal angle nearly right-angled (Fig. 8d). Veins brownish yellow. Venation: Sc ending before fork of Rs, at about 5/6 of Rs; basal section of R_5_ about 1½ as long as r-m; m-cu shortly before fork of M, distance approximately 1/3 its own length; basal section of M_3_ about twice as long as m-m; cell m_1_ longer than cell dm. Halter pale with stem light yellow.

***Abdomen*.** Tergites 1–6 brown, tergites 7 and 8 dark brown. Sternites 1–6 brownish yellow; sternites 7 and 8 dark brown.

**Figure 9.**
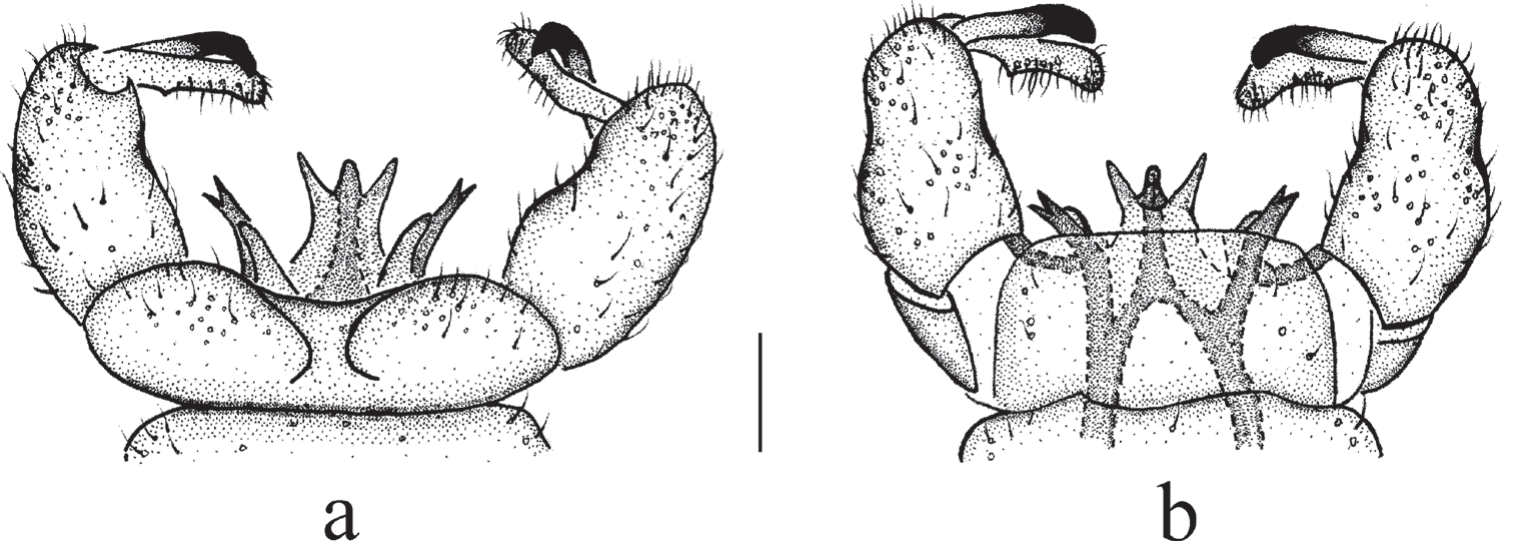
Antocha (Antocha) tibetana sp. nov. **a** male hypopygium, dorsal view **b** male hypopygium, ventral view. Scale bar: 0.1 mm.

***Hypopygium*** (Figs 8e, 9). Yellow. Posterior margin of tergite 9 with shallow emargination (Fig. 9a). Gonocoxite nearly cylindrical with brown setae (Figs 8e, 9). Outer gonostylus apically black with tip curved, claw-shaped. Inner gonostylus nearly straight with tip rounded. Interbase nearly V-shaped, distal part flattened and horn-like, with tip blunt (Figs 8e, 9). Paramere with base rod-shaped; apical part flattened, triangular in shape. Inner branch of paramere with tip bifid, two teeth almost equal in length (Figs 8e, 9). Aedeagus rod-shaped, curved ventrally (Figs 8e, 9).

**Female.** Body length 5.0–5.3 mm, wing length 5.5–5.7 mm. Generally similar to male by body coloration.

***Ovipositor*** (Fig. 8f). Tergite 10 yellowish. Cercus pale yellow, with base darker; tip raised and tapering. Hypogynial valve yellowish, reaching approximately middle of cercus.

##### Etymology.

The species is named after the type locality, Tibet.

##### Distribution.

China (Tibet).

##### Remarks.

The new species is somewhat similar to A. (A.) spiralis from China and India with the similar wing venation and bifid tip of inner branch of paramere, but it can be easily distinguished by the three stripes on the thorax (Fig. 8c) and the triangular apex of the paramere (Figs 8e, 9). In A. (A.) spiralis, the thorax has no obvious longitudinal stripes (Fig. 6c), while the apex of the paramere is slender and twisted into a spiral (Figs 6e, 7).

## Supplementary Material

XML Treatment for Antocha (Antocha) curvativa

XML Treatment for Antocha (Antocha) setigera

XML Treatment for Antocha (Antocha) spiralis

XML Treatment for Antocha (Antocha) tibetana
